# Personality in juvenile Atlantic cod ecotypes and implications for fisheries management

**DOI:** 10.1002/ece3.9952

**Published:** 2023-04-19

**Authors:** Rosanne Beukeboom, Joseph S. Phillips, Guðbjörg Ásta Ólafsdóttir, David Benhaïm

**Affiliations:** ^1^ Research Centre of the Westfjords University of Iceland Bolungarvik Iceland; ^2^ Department of Aquaculture and Fish Biology Hólar University Saudárkrókur Iceland; ^3^ Department of Biology Creighton University Omaha Nebraska USA

**Keywords:** Atlantic cod, behavioral syndrome, management, migration, *Pan* I, personality

## Abstract

Animals show among‐individual variation in behaviors, including migration behaviors, which are often repeatable across time periods and contexts, commonly termed “personality.” These behaviors can be correlated, forming a behavioral syndrome. In this study, we assessed the repeatability and correlation of different behavioral traits, i.e., boldness, exploration, and sociality, and the link to feeding migration patterns in Atlantic cod juveniles. To do so, we collected repeated measurements within two short‐term (3 days) and two long‐term (2 months) intervals of these personality traits and genotypes of the *Pan* I locus, which is correlated with feeding migration patterns in this species. We found high repeatabilities for exploration behavior in the short‐ and long‐term intervals, and a trend for the relationship between exploration and the *Pan* I locus. Boldness and sociality were only repeatable in the second short‐term interval indicating a possible development of stability over time and did not show a relation with the *Pan* I locus. We found no indication of behavioral syndromes among the studied traits. We were unable to identify the existence of a migration syndrome for the frontal genotype, which is the reason that the link between personality and migration remains inconclusive, but we demonstrated a possible link between exploration and the *Pan* I genotype. This supports the need for further research that should focus on the effect of exploration tendency and other personality traits on cod movement, including the migratory (frontal) ecotype to develop management strategies based on behavioral units, rather than treating the population as a single homogeneous stock.

## Graphical abstract

By collecting repeated behavioral measurements of exploration, boldness, and sociality and the feeding migration‐linked *Pan* I genotype, we studied the link between personality and feeding migration in Atlantic cod juveniles. The results show that personality is apparent and that exploration shows a trend to be linked to the *Pan* I locus, which might have implications for fisheries management.

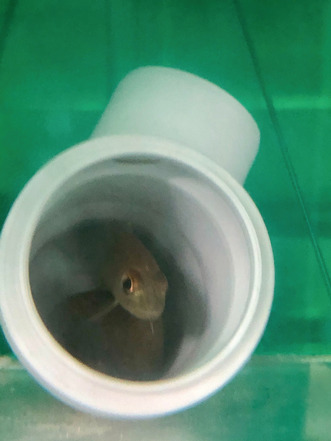

## 1 INTRODUCTION

Animals show among‐individual variation in behaviors, which when repeatable across time periods and contexts are commonly termed “personality” (Dingemanse & Wright, 2020). Personality can significantly influence life history, ecology, and evolution (Biro & Stamps, 2008; Wolf & Weissing, 2012), thereby acting on individual, population, and ecosystem levels (Bolnick et al., 2011; Cote et al., 2017; Hunter Jr et al., 2021; Mittelbach et al., 2014; Réale et al., 2010). One such example is personality in migration behavior (Bowler & Benton, 2005), where migration is defined as a (usually seasonal) round‐trip (Stenseth & Lidicker, 1992).

Personality‐based migration patterns can lead to personality‐related differences in individual life history, such as growth rate and survival (Clobert et al., 2009) because time and energy are spent on dispersion, rather than on foraging or immunity but with the benefit of the possible discovery of better feeding grounds (Raffard et al., 2022). Furthermore, an optimal mix of personalities in a population can influence its survival by supporting the different stages (departure, translocation, and settlement) differently (Bowler & Benton, 2005; Cote, Clobert, et al., 2010). For example, bolder individuals might be more successful in the decision to depart (Bevan et al., 2018), more explorative individuals might be better in the translocation, and less aggressive individuals might thrive better in settlement (Duckworth & Badyaev, 2007). Finally, behavioral differences in migration can influence ecosystems in a variety of ways, such as personality‐related prey abundance reduction (Cote et al., 2017; Raffard et al., 2022) and seed migration abilities (Snell et al., 2019; Zwolak & Sih, 2020).

Réale et al. (2007) provided a terminology for studying personality by defining five broad categories (i.e., shyness‐boldness, exploration‐avoidance, activity, sociability, and aggressiveness). Out of these five categories, three are often found to be correlated to migration (Coates et al., 2019; Cote et al., 2017; Cote, Fogarty, et al., 2010), i.e., *shyness‐boldness*: “an individual's reaction to any risky, but not new situation,” *exploration‐avoidance*: “an individual's reaction to a new situation” and *sociability*: “an individual's reaction to the presence or absence of conspecifics, excluding aggressive behavior” (Réale et al., 2007). These traits all act on the decisions of whether, when, and where an individual should migrate (Shaw, 2020). For example, bolder great tits (Dingemanse et al., 2003) and killifish (Fraser et al., 2001) dispersed over greater distances than shyer individuals. Bolder wild elk (Found & St. Clair, 2019), lizards (Damas‐Moreira et al., 2019), and common roach (Chapman, Hulthén, et al., 2011) were more likely to disperse than their shyer counterparts. More explorative lizards (Damas‐Moreira et al., 2019; Michelangeli et al., 2017), voles (Hoset et al., 2011), and butterflies (Reim et al., 2018) dispersed further than less explorative individuals. Finally, asocial mosquitofish (Cote et al., 2017) and yellow‐bellied marmots (Blumstein et al., 2009) dispersed further than social individuals and asocial lizards dispersed earlier with higher population densities (Cote & Clobert, 2007).

Boldness, exploration, and sociality are often correlated, where higher levels of boldness and exploration and lower levels of sociality are positively related to migration occurrence and distance, forming a migration syndrome (Bevan et al., 2018; Clobert et al., 2009; Coates et al., 2019; Comte & Olden, 2018; Nilsson et al., 2014). These syndromes can be underpinned by physiological traits, e.g., hormone levels and corresponding genes (Réale et al., 2007), life history, and morphological traits, but the latter two have shown to be of less importance (Dingle, 2006). Correlated behaviors limit plasticity and might constrain animals in their ability to behave optimally in every situation (Conrad et al., 2011). This means that personality traits ideally should not be studied without consideration of other traits, because it could only reveal the cost or benefit of the behavior in a particular context while ignoring the possible influence of other traits of equal importance, which carry their own costs and benefits (Sih, Bell, & Johnson, 2004).

The Atlantic cod (*Gadus morhua*) is an ideal candidate for studying the theory of personality traits influencing migration, leading to differences in life history and population structure, with potential implications for management. This species is widespread throughout the continental shelf on both sides of the North Atlantic Ocean and is an apex predator, which makes it a key species in its ecosystem (Link & Garrison, 2002). Although the Atlantic cod has few natural predators, it is of high commercial importance and overfishing has reduced the worldwide population size by as much as 99.9% of its historical levels between 1960 and 1990 (Christensen et al., 2003; Hutchings & Reynolds, 2004). Management measures ranging from a total fishing ban to size‐dependent fishing quotas have given the species some space to start a slow recovery, moving away from “critically endangered” to “vulnerable” on the IUCN red list (Sobel, 1996). However, current measurements that are often based on biomass estimates of an assumed homogenous genetic and/or phenotypic group of individuals (Kerr et al., 2017), are not everywhere as successful, as the recovery has been slow or even nonexistent in some populations (Hutchings & Reynolds, 2004). This leads to the search for other factors than biomass as facilitators of recovery, such as behavioral mechanisms, i.e., the transfer from quantitative to qualitative quota (Olsen et al., 2012; Petitgas et al., 2010).

In Iceland, the cod population has been fairly stable since 2002 when quotas were established, but a recent reduction in population size has been observed (MFRI Assessment Reports, 2020). The life cycle of the Icelandic cod starts as larvae on a multitude of spawning grounds spread around the country (Marteinsdottir et al., 2000; Sólmundsson et al., 2015). Part of the larvae drift off to deeper waters, but most of them drift to shore where they settle for the first four years of life. After four years, the individuals begin to show nonbreeding partial migration (Chapman, Brönmark, et al., 2011). “Residents” or “coastal cod” perform feeding migrations close to the shore year‐round, while “migrants” or “frontal cod” make more extensive feeding migrations (100–1000 km) outside the spawning season (June–January) (Pálsson & Thorsteinsson, 2003). These migration differences result in differences in habitat use by the two ecotypes during the feeding season; coastal are mainly found in water that is shallower (<200 m) and warmer (x̄ 7.3°C) water than where frontal reside (200–600 m; x̄ 4.6°C) (Pálsson & Thorsteinsson, 2003; Robichaud & Rose, 2004; Thorsteinsson et al., 2012). Consequently, these differences in habitat use during the feeding season by the two ecotypes have led to differences in life history traits (Jónsdóttir et al., 2008; McAdam et al., 2012). Interestingly, both the coastal and the frontal migration patterns are consistent over multiple years, i.e., coastal fish remain close to shore, while the frontal always performs its long‐distance feeding migration (Thorsteinsson et al., 2012).

The two ecotypes do not only differ in behavior but also possess different morphological (McAdam et al., 2012) and genetic characteristics, such as the ancient evolutionary stable pantophysin locus (*Pan* I; Kirubakaran et al., 2016; Pampoulie et al., 2008; Pogson & Mesa, 2004). The *Pan* I locus codes for an integral membrane protein, which is expressed in cytoplasmic transport vehicles (Windoffer et al., 1999). The function related to the behavioral ecotypes remains unclear, but the gene resides in a supergene (Linkage group 1, LG1; Matschiner et al., 2022; Pampoulie et al., 2022), comprising the rhodopsin gene related to dim light perception (Andersen et al., 2015; Berg et al., 2016; Pampoulie et al., 2015), and genes encoding for hemoglobin‐induced temperature preference (Petersen & Steffensen, 2003), brain function and potentially swim bladder function (Kirubakaran et al., 2016). In the Icelandic cod population, the distribution of individuals carrying the *Pan* I^AA^ genotype is highly skewed towards the coastal behavioral ecotype, while individuals carrying *Pan* I^BB^ are skewed towards the frontal behavioral ecotype (Pampoulie et al., 2008), although a recent study showed that the *Pan* I^AA^ is a better indicator for residency than *Pan* I^BB^ is for migratory behavior (Pampoulie et al., 2022). The behavior of heterozygotes (*Pan* I^AB^) is ambiguous: they have been shown to behave like either of the homozygotes (Pampoulie et al., 2008), show coastal behavior (Árnason et al., 2009), or differ from coastal behavior (Beukeboom et al., 2022).

Given the evidence that personality can be linked to migration, that Atlantic cod show different migration patterns and the existence of unanswered questions regarding population management, this study focuses on unraveling the link between personality and migration in Atlantic cod juveniles. Studying juveniles specifically allows for investigating the initiation and development during the early life stages (Petitgas et al., 2010; Polverino et al., 2016). As juveniles might face different challenges than adults, personality change can be expected during ontogeny, i.e., developmental plasticity, which can influence behaviors expressed later in life (Bowler & Benton, 2005; Polverino et al., 2016; Schuster et al., 2017; Stamps & Groothuis, 2010). So far, most studies including *Pan* I have focused on adults (Fevolden et al., 2012), while juveniles are important for the recruitment of the Icelandic stock (Jonasson et al., 2009).

To get insight into the link between personality and migration, we measured personality over short‐ (3 days) and long‐term (2 months) intervals, aiming to answer the following specific research questions: (1) Do Atlantic cod juveniles show consistent behavioral individual differences for exploration, boldness, and sociality, i.e., personality, in the short‐term (3 days) and long‐term (2 months)?; (2) Do these behavioral differences correlate, forming a behavioral syndrome? (3) Can *Pan* I be integrated forming a migration syndrome together with these personality traits, i.e., are cod carrying the *Pan* I^BB^ more explorative, bolder, and more (a)social than cod carrying the *Pan* I^AA^?

We predicted that Atlantic cod juveniles show consistent individual differences in bold, explorative, and sociality behavior within short‐ (3 days) and long‐term (2 months) intervals, as juvenile Atlantic cod have shown differences in other behaviors, that these behaviors are repeatable and that this continues into adulthood (Beukeboom et al., 2022; Hansen et al., 2008; Hart & Salvanes, 2000; Villegas‐Ríos et al., 2018; Zimmermann et al., 2012). We predicted that juvenile cod show a behavioral syndrome, where a higher boldness and exploration and lower sociality are correlated as already shown in adult cod and other fish species (Coates et al., 2019; Cote, Fogarty, et al., 2010). Finally, we predicted that the migration type, identified by *Pan* I genotypes, can be integrated forming a migration syndrome, where cod carrying the *Pan* I^BB^ are bolder, more explorative, and less social, while cod with the *Pan* I^AA^ are shyer, less explorative and more social. This assumption is based on the link between *Pan* I and migration behavior (Pampoulie et al., 2008, 2022; Thorsteinsson et al., 2012) and that exploratory behavior has a genetic basis in adult cod (Drangsholt et al., 2014). Getting insight into these questions will increase our knowledge about how personality could be linked to susceptibility to the harvesting of Atlantic cod and thereby provides input for population management tools.

## 2 METHODS

The methods followed the same protocol as described in Beukeboom et al., 2022; 102 age 0+ cod juveniles (weight range = 0.75–4.39 g and mean = 1.87 g; standard length range = 3.83–7.55 cm and mean = 5.72 cm) were beach seined from the 3rd to 12th of October 2019 in three different fjords around the Westfjords of Iceland to obtain genetical variation. They were transported to a laboratory in Bolungarvik, Iceland, and housed individually in 9.5‐L tanks (~29 × 21 × 19 cm, water level 16 cm, Aquaneering Inc.). The recirculating system contained freshwater mixed with marine salt to achieve a natural salinity of 30 ± 2‰, a temperature of 11 ± 1°C (November) and 10 ± 1°C (December–June), ammonia levels of <0.5 ppm, oxygen levels of 10.4 ± 0.1 mg/L and a constant photoperiod of 12 h:12 h (7 AM–7 PM GMT). The water circulated through the Aquaneering system, passing through all the tanks, a biofilter, sieves (mesh size 25 μm), and a UV light for sterilization. Every tank had a gray PVC pipe to provide shelter to the fish. Fish were fed twice a day alternating defrosted shrimp and bloodworms daily ad libitum. On the experimental days, feeding took place after the experiment to avoid any differences in feeding motivation. Every month, after the first trial of the open field experiments (see below), the fish were measured for weight and standard length. In November, a set of trial experiments was performed, which are presented in Beukeboom et al., 2022.

### 2.1 Behavioral tests

**FIGURE 1:**
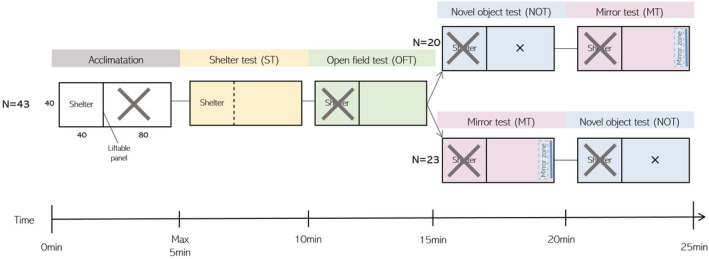
Experimental setup of the four different tests. Dimensions are in centimeters, crossed areas are inaccessible during the experiment.

Of the initial 102 caught individuals, 43 were included in the analysis (see Results). Each individual underwent a cycle of a shelter test (ST, boldness), open field test (OFT, exploration), novel object test (NOT, boldness), and mirror test (MT, sociality) during four trials: January 19–21 (Trial A), January 22–24 (Trial B), March 15–17 (Trial C), and March 18–20 (Trial D) (Figure 1). All fish started with the ST, followed by the OFT. Consecutively, 20 fish were subjected to first the NOT and then the MT, while the rest (*N* = 23) received the MT first and then the NOT to take any influence of the NOT or MT test order into account (Figure 1). In addition, all fish were tested in the same overall order to standardize the intervals between the tests for each fish. Data in the ST was collected manually, while OFT, NOT, and MT videos were analyzed with video‐tracking software (Ethovision, Noldus, v. 15.0). A smoothing parameter of 0.1 was set in the video‐tracking software, which set the sample points to the previous location until the distance moved was more than 0.1 cm, which removed any noise of moving pixels due to imperfect light conditions, as this was found to be appropriate for the setup we used (Beukeboom et al., 2022). The water temperature of the experimental tank ranged from 9.2 to 11.5°C.

### 2.2 Shelter test (ST)

The shelter test is commonly used as a measure of boldness (see for an overview Toms et al. (2010)), where bolder fish have a shorter exit time. The fish was gently captured with a dipnet out of its home tank and placed in a shelter (26 L; 40 × 40 × 40 cm, water level 16 cm) and after five minutes of acclimation, the door was lifted. The fish was given 5 min to leave the shelter, and the latency to exit was recorded. If it did not leave voluntarily, it was gently forced out using a dip net into the arena (51 L; 80 × 40 × 40 cm, water level 16 cm) and given a maximum score of 300 s. Unfortunately, in 95 out of 158 ST trials (60.1%), fish did not leave the shelter voluntarily. This was most likely due to the test duration being too short, which resulted in insufficient variation in this behavior. This original boldness measurement was therefore excluded from further analysis, but a binary factor (left shelter y/n) was included in our models to account for any influence of the fish being pushed out of the shelter rather than leaving voluntarily.

### 2.3 Open field test (OFT)

In absence of a predator, juvenile cod have been shown to move freely across an open space (Nordeide & Svåsand, 1990), which most likely serves the function of information gathering (Hughes, 1997). Therefore, the OFT was used as a proxy for exploration, where it is expected that explorative fish swim greater distances and cover bigger areas than nonexplorative individuals. The test was carried out as follows: as soon as the fish entered the arena during the ST (51 L; 80 × 40 × 40 cm, water level 16 cm, Figure 1), the shelter was closed and the fish was video recorded for five minutes. The total distance traveled and the total area covered (i.e., traversed, calculated by using the total unique x/y coordinates rounded to the nearest integer) were extracted. Area covered and distance traveled (both log‐transformed) were highly correlated (PEARSON: *r* = 0.97; CI 95% [0.96–0.98]; *p* < 0.001). Because the area covered is statistically ceiled (i.e., there is a maximum number of unique X/Y coordinates available), the total distance traveled was used in further analysis, to catch the maximum variation possible.

### 2.4 Novel object test (NOT)

The novel object test was used as a measure of boldness, where shy individuals are expected to flee, retreat, or freeze, while bold individuals are expected to become more active and approach the novel object (Toms et al., 2010). The NOT was carried out after the OFT (*N* = 20) or after the MT (*N* = 23). When present, the mirror was removed, and a novel object was dropped in the middle of the arena (Figure 1). In January, this was a red, tin can (⌀ 6 cm, 113 cm^2^) and in March this was a blue plastic pipette tip rack (13 × 10 cm; 117 cm^2^) to reduce habituation to the object. Five minutes were video recorded and the mean distance to the object was extracted as a proxy for boldness.

### 2.5 Mirror test (MT)

Cod juveniles can show plastic social behavior, from shoaling to aggression (Meager et al., 2018). The mirror test can be used to measure both sociality and aggression, depending on the species and developmental phase. While Villegas‐Ríos et al. (2018) used the mirror test as a measure of aggression in adult cod, the life stage where the risk of being predated is minimal, we assume that the mirror test in this study on juveniles elicited social behavior instead for two reasons. Firstly, the arena was open with no shelter to hide, which has been shown to elicit shoaling behavior in juvenile cod (Laurel et al., 2004). Secondly, aggressive/submissive behavior often occurs when opponents differ in size (McCormick & Weaver, 2012; Sverdrup et al., 2011), and as the mirror just reflects the fish itself, these size differences are nonexistent. The MT was carried out after the OFT (*N* = 20) or after the NOT (*N* = 23). When present, the object was removed, and a mirror was placed opposite the shelter door (Figure 1). Five minutes were video recorded, and the total time spent in a 10 cm zone in front of the mirror was extracted as a proxy of sociality. After the experiment, visual inspection of the videos for aggressive and/or social behaviors confirmed our decision; social behaviors, such as repeated approaches to the mirror at cruising speed and “hanging around” the mirror were highly represented, and aggressive behaviors such as accelerations towards the mirror, biting or c‐shaping were absent (Sverdrup et al., 2011).

### 2.6 Genotyping

In June 2020, at the end of the overall project, the fish were euthanized with phenoxyethanol (1.6 mg/L) and fin clips were taken to assign the fish to either coastal (*Pan* I^AA^), frontal (*Pan* I^BB^), or heterozygote (*Pan* I^AB^) using PCR analyses as described in Pampoulie et al. (2006).

### 2.7 Statistics

**FIGURE A1:**
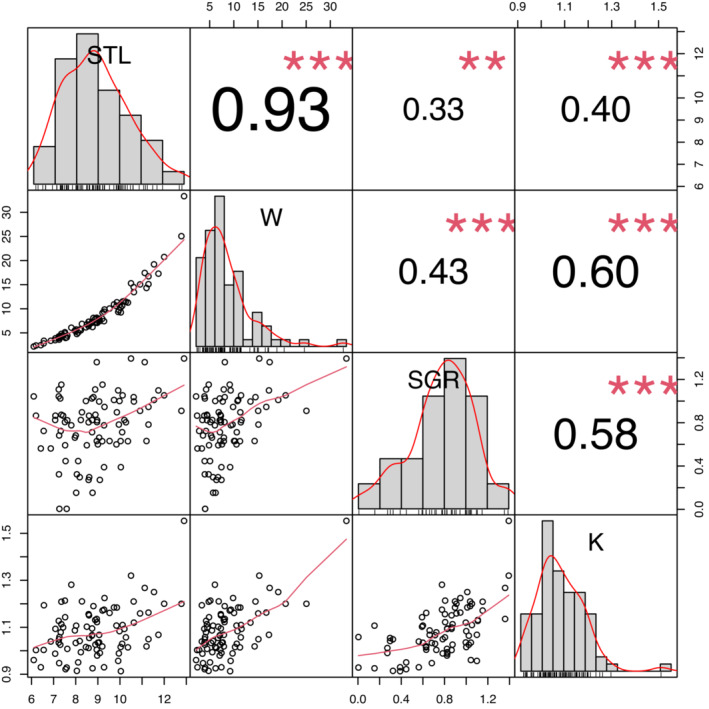
Pearson correlation matrix of possible effects on the personality estimates.

All data were analyzed using the same method as in Beukeboom et al. (2022) using R (v. 4.1.2; R Core Team, 2021). We fit multivariate linear mixed models to estimate the repeatabilities of and the correlations between the total distance traveled (OFT), mean distance to object (NOT), and time spent in the mirror zone (MT). The models were fit using the Bayesian software Stan (Carpenter et al., 2017) run via the “brms” package (Bürkner, 2017). We ran four separate models containing four different subsets of the data: short‐term (∆ 3 days) for January (trials 1 and 2; SJ) and March (trials 3 and 4; SM) and long‐term (∆ 2 months) for the first trials (trial 1 and 3; LA) and the second trials (trial 2 and 4; LB) of each month. The models simultaneously regressed each dependent variable (i.e., OFT, NOT, and MT estimates) against a set of fixed and random effects while also quantifying the covariance between the dependent variables. The fixed effects of weight (g), standard length (cm), Fulton's condition factor (*K* = *Weight*/*Length* ^3^ × 100), and specific growth rate (*SGR =* ∆ ln(weight) *100/∆ day) that could influence the personality estimates were evaluated for collinearity. Pearson correlations revealed that all measurements were substantially correlated (Figure A1) and therefore only SGR was included in the analysis. The three personality estimates and SGR were scaled using z‐scoring (subtracted the mean and divided by the standard deviation) separately for the subset of data used in each model. The full version of each model was fit with the scaled personality measurement as response variables (i.e., total distance traveled, mean distance to object, and total time in the mirror zone), the four fixed effects of genotype, SGR (since the previous month for short‐term, January–March for long‐term), trial and shelter leave (y/n). We include a binary covariate to indicate the order in which fish were tested for the NOT and MT. The random‐effects structure included individual fish identity (ID) as a grouping variable, allowing us to calculate the repeatability of the personality estimates as the ratio of the among‐individual variance and the sum of the among‐individual and residual‐level variances (Johnson & Koch, 2011). Moreover, the model estimated covariances between the personality estimates at both the ID and residual levels. The among‐individual covariance quantified the degree to which the personality estimates were correlated among individuals across multiple trials (i.e., behavioral syndrome), while the residual level covariance quantified the degree to which the personality estimates were correlated among observations independently of the identity of individuals. The model was run for 4000 iterations (2000 for warmup and 2000 for sampling), four chains, an adapted delta of 0.9, and all other parameters set to their defaults. Convergence was assessed using the standard diagnostics provided by Stan (Bürkner, 2017; Carpenter et al., 2017), including the potential scale reduction factor ($\hat{R}$), effective sample size, and visual inspection of trace plots and histograms for each model parameter. We used medians for point estimates and quantiles with 95% coverage for uncertainty intervals (UI_95%_).

### 2.8 Ethical note

The number of fish and the procedures (fishing, handling, fin‐clipping, and behavioral tests) were chosen to adhere to strict ethical guidelines, but an ethics committee approval for the research project was not required by Icelandic regulation (Act No. 55/2013 on Animal Welfare).

## 3 RESULTS

The 102 0+ juvenile cod caught in October 2019 consisted mainly of coastal cod (*Pan* I^AA^; *N* = 75), with substantially fewer heterozygotes (*Pan* I^AB^; *N* = 17) and very few migratory individuals (*Pan* I^BB^; *N* = 4). The *Pan* I locus of six fish could not be determined due to failing analysis. A high mortality rate (*N* = 56) caused by the incapability of adjusting to the laboratory food, which is not uncommon for the transfer from the wild to the lab, caused a major reduction in the sample size. Additionally, the individuals with unknown genotypes, fish that lost weight between January and March, and fish that had a condition factor below the mortality threshold of 0.8 (Marteinsdottir & Begg, 2002), were removed. This left only one individual with the migratory genotype. Therefore, we only analyzed data from the coastal and heterozygotes, comprising a final dataset of 43 fish. Although all fish performed the experiments, which should have led to 540 observations (43 fish * 3 tests * 4 trials; 90 per subset), video failures reduced the number to 328 trials (83 observations in the models SJ, SM, and LA; 79 in LB). The 43 individuals gained weight from the start to the end of the experiments (January–March) from x̅ 6.48 ± SD 3.07 (start) to 10.05 ± 6.18 gr (end), had a mean Fulton's condition factor of 1.08 ± 0.10 (start) and 1.09 ± 0.12 (end) and a specific growth rate of 0.78 ± 0.30% body weight/day (start‐end). Thirty‐five were assigned as coastal (*Pan* I^AA^) and eight were heterozygotes (*Pan* I^AB^). All models converged with $\hat{R}$ = 1, had well‐mixed chains, and no extreme trails were visible in the trace plots. The total distance traveled ranged from 49.2 to 4147.7 cm (x̅ = 1318.2 cm), the mean distance to the object ranged from 14.2 to 36.6 cm (x̅ = 25.7 cm) and the total time in the mirror zone from 0.0 to 282.1 s (x̅ = 82.8 s).

### 3.1 Repeatabilities

**FIGURE 2:**
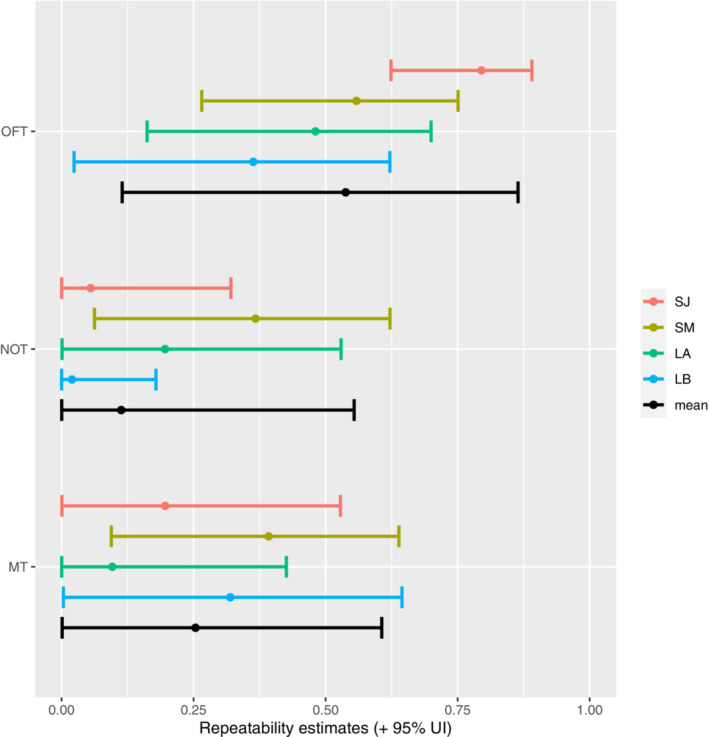
Repeatabilities and their 95% UI short‐term (three days) intervals within January (SJ) and March (SM) and between two months (long‐term) intervals for the first and second trials (LA; LB) and their combined mean (black) for exploration (OFT), boldness (NOT) and sociality (MT). Estimates with a median away from zero and error bars non‐bordering 0 are considered repeatable.

**FIGURE 3:**
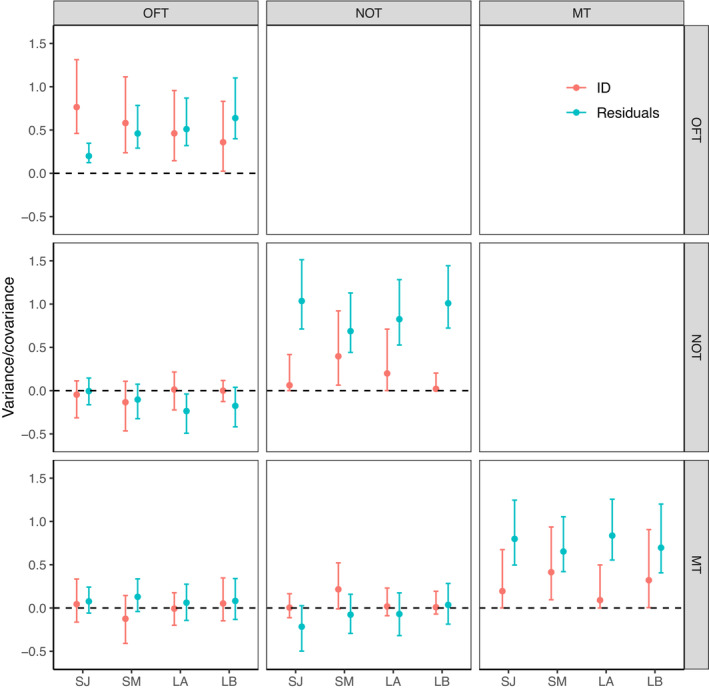
Medians and their corresponding 95% uncertainty intervals for the variances and pairwise covariances of exploration (OFT), boldness (NOT), and sociality (MT) for the two short‐term (3 days; SJ and SM) and long‐term (2 months; LA and LB) models on both the ID (red) and residual level (blue). The dashed line indicates zero.

**FIGURE 4:**
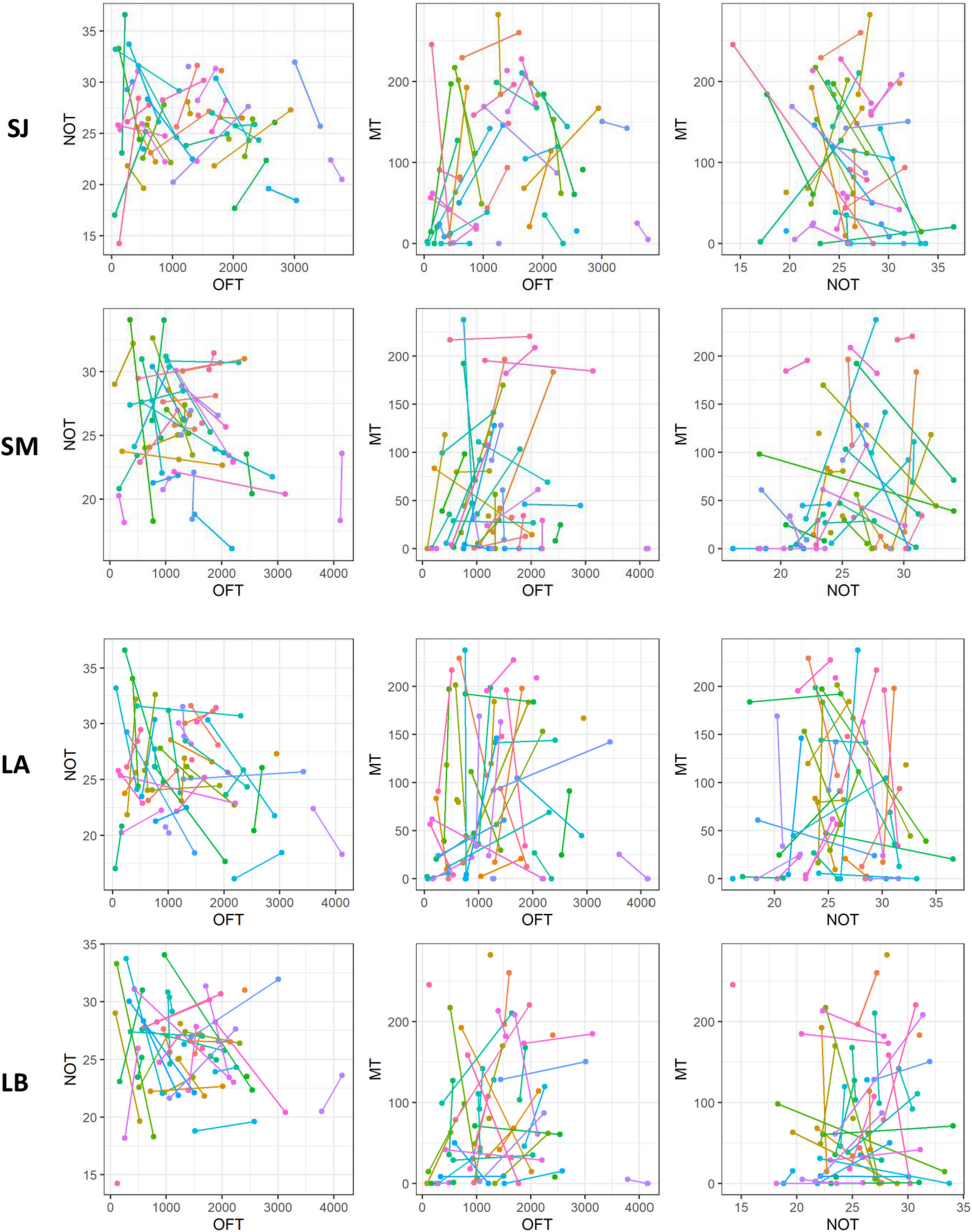
Pairwise combinations of exploration (OFT), boldness (NOT), and sociality (MT) on the short‐term for January (SJ) and March (SM) and long‐term correlation for trials A (LA) and B (LB) of January and March. Connected/colored dots are estimates for the same individual. Within axis distance (i.e., short or long) indicates repeatability, overall dot pattern (i.e., decreasing/increasing or no pattern) indicates a behavioral syndrome and the direction of the lines (i.e., similar or different) indicates a correlation on the residual level.

Among‐individual variance (ID) of total distance traveled in the OFT (i.e., exploration) was unambiguously different from zero both in the short‐term and long‐term models, indicating individual repeatability for this trait. Among‐individual variance exceeded the within‐individual (residual) variance in the short‐term intervals, such that the behavior was highly repeatable (January: R = 0.80; UI_95%_ = [0.62, 0.89]; March: R = 0.56; UI_95%_ = [0.27, 0.75], Figures 2, 3, 4, Table A1). By contrast, the residual variance exceeded the among‐individual variance in the long‐term intervals, resulting in lower repeatability (Trial A: R = 0.48; UI_95%_ = [0.16, 0.70]; Trial B: R = 0.36; UI_95%_ = [0.02, 0.62], Figures 2 and 3, Table A1). Repeatabilities for mean distance to object in the NOT (i.e., boldness) and time spent in front of the mirror in the MT (i.e., sociality) was overall low, although in both cases, the short‐term interval in March was repeatable (Table A1). This increase in repeatability between the short‐term models of January and March was mainly caused by an increase in the among‐individual variance and to a lesser extent a decrease in the residual variance (Figures 2 and 3, Table A1). These patterns are also visible in the raw data, where OFT has short lines on its axis and NOT and MT long lines (Figure 4).

### 3.2 Behavioral syndromes

Covariances of the pairwise combination of the three estimates (OFT‐NOT, NOT‐MT, and OFT‐MT) for all four models (SJ, SM, LA, and LB) were all close to zero with UI_95%_ strongly overlapping zero (Figure 3, Table A2). Additionally, no visible association was apparent on the ID level (no apparent increasing or decreasing dot patterns), nor on the residual level (lines not pointing in the same direction) when plotting all combinations of the personality traits pairwise (Figure 4). These results show no evidence of a behavioural syndrome between any of those traits.

### 3.3 Fixed effects

**FIGURE 5:**
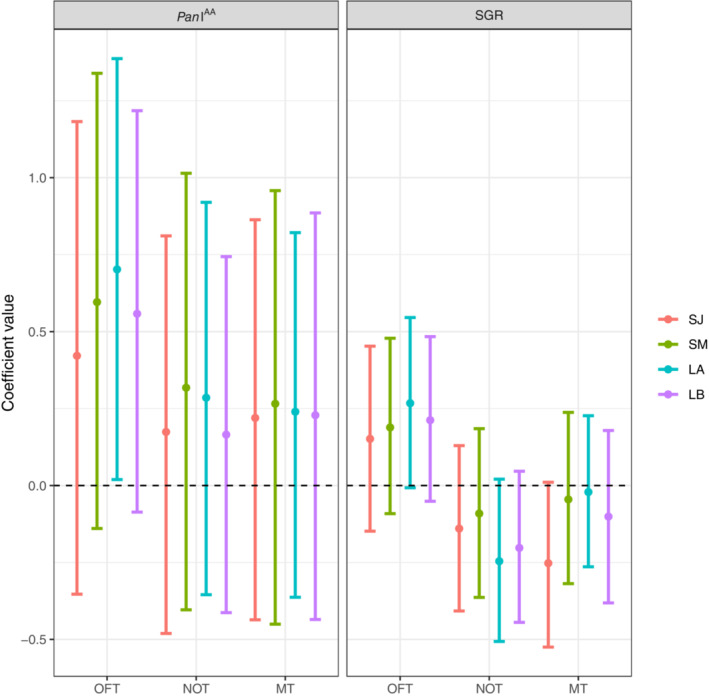
Medians and their corresponding 95% uncertainty intervals for the fixed effect of Pan I genotype and specific growth rate (SGR) for exploration (OFT), boldness (NOT), and sociality (MT) for the two short‐term (SJ and SM) and long‐term (LA and LB) models. The dashed line indicates zero. Because SGR was z‐scored, the co‐efficient values are presented in units of standard deviation.

**FIGURE A2:**
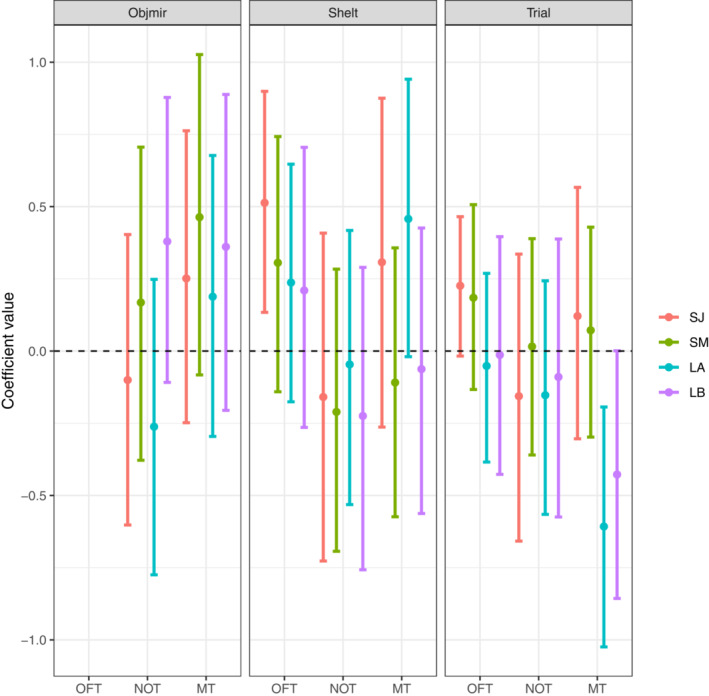
Median and 95% uncertainty intervals of the covariances of the four models (SJ, SM, LA and LB) between the pairwise personality measurements (OFT, NOT and MT).

The *Pan* I genotype shows a trend to influence the total distance traveled during the OFT, with the most uncertainty in the short‐term January and the least in the long‐term first trial: coastal individuals tended to move more than heterozygotes. The *Pan* I genotype did not show to influence the mean distance to object, nor the total time spent in the mirror zone (Figure 5, Table A2). A trend was also visible for specific growth rates in the OFT as all estimates are away from zero but with slightly overlapping UI_95%_. Fish with a higher SGR might travel longer distances during the OFT. A similar trend was visible for the NOT; faster‐growing fish were on average closer to the object, with the strongest evidence for this in the long‐term models where point estimates and uncertainty intervals were farther from zero. A final trend was visible for the MT; fish with a higher SGR spent less time in the mirror zone in the short‐term interval in January, but no influence was visible in the other models, where point estimates were close to zero with strongly zero‐overlapping uncertainty intervals (Figure 5, Table A2). The order of the experiments, shelter leave, and trial number had no clear effects on the personality traits (Table A2, Figure A2).

## 4 DISCUSSION

In this study, we aimed to examine the link between personality and migration tendencies, using the migration‐linked *Pan* I locus in Atlantic cod juveniles. To do so, we collected repeated measurements within short‐ (3 days) and long‐term intervals (2 months) of exploration, boldness, and sociality and genotypes for the *Pan* I locus. Using this data, we aimed to answer the following research questions: (1) Do Atlantic cod juveniles show personality over short‐ (3 days) and long‐term (2 months) intervals for boldness, exploration, and sociality?; (2) Do these behavioral differences correlate into behavioral syndromes? (3) Can *Pan* I be integrated to form a migration syndrome with these personality traits? We found that exploration behavior was repeatable in the short‐ and long‐term intervals, with a possible link with the *Pan* I locus, where coastal fish might be more explorative than heterozygotes. By contrast, boldness and sociality were only repeatable in the second short‐term interval. Moreover, the personality estimates were not correlated to each other at the individual level, indicating the lack of any behavioral syndrome. We were unable to identify the existence of a migration syndrome for the frontal genotype, which is the reason that the link between personality and migration remains inconclusive, but we demonstrated a possible link between exploration and the *Pan* I genotype, which supports the need for further research on this topic.

### 4.1 Personality in Atlantic cod juveniles

Exploration was found to be repeatable between three‐day intervals but also between two months. Although it is difficult to compare repeatabilities across studies with different time intervals between trials(Biro & Stamps, 2008), the short‐term repeatability found in this study is higher than that was found in a study on adult Atlantic cod (Villegas‐Ríos et al., 2018) and the long‐term repeatability is similar to a previous study on cod juveniles at an earlier age (Beukeboom et al., 2022). The higher repeatabilities found for the short‐term compared with the long‐term intervals in this study for exploration were also found in a meta‐analysis (Bell et al., 2009). This supports the finding that cod show consistent individual differences in exploration behavior, i.e., personality both at juvenile and adult stages whereas boldness and sociality seem to be more plastic at this developmental stage. Both were only repeatable in the short‐term interval in March, mainly caused by an increase in the among‐individual variance and to a lesser extent to a decrease in the within‐individual variance compared with January. This indicates that boldness and sociality strengthen with fish age as was found in cichlids (Budaev et al., 1999) and mosquitofish (Polverino et al., 2016), but further research over a longer time period is needed.

### 4.2 Behavioral/migration syndrome

This study found no evidence that exploration, boldness, and sociality were correlated in Atlantic cod juveniles and thereby not indicating a full‐suit behavioral syndrome for migration, as was found in Nilsson et al. (2014). The lack of a behavioral syndrome could be explained by environmental changes in cod natural habitat that might favor the plasticity of these traits (Sih, Bell, Johnson, & Ziemba, 2004). Further research with a larger sample of individuals, including frontal individuals, is needed to confirm this result (Garamszegi et al., 2012).

Of the three personality traits measured, only exploration showed a tendency of being influenced by the *Pan* I locus in both the short‐ and long‐term intervals, where coastal individuals traveled greater distances than heterozygotes and therefore are believed to be more explorative. Interestingly, in a previous study, the same pattern was found at an earlier stage of development i.e., up to five months earlier (Beukeboom et al., 2022). This strengthens the support for a stable link between personality, development, and the migration‐linked *Pan* I locus in Atlantic cod. Moreover, this correlation in combination with the high repeatability of exploration gives support to the idea that exploration has a heritable basis as was found in adult cod (Drangsholt et al., 2014) and thereby has the potential to form a basis of personality‐based evolution (Bell, 2008; Dochtermann et al., 2015). There is no current evidence for a relationship between exploratory behavior and heritability in other fishes (e.g., brown trout: Kortet et al. (2014); zebrafish: Lamb (2018)), but a genetic basis was found in great tits (Drent et al., 2003; Mouchet et al., 2021).

Due to a lack of the migratory genotype in our study, we could not link personality to the *Pan* I locus for this ecotype as intended and are therefore unable to draw any conclusions about the link between personality and migration. We also could not study how coastal and heterozygotes behave compared with the frontal ecotype. It is possible that a behavioral syndrome is only detectable when correlating coastal with frontal cod, which remains to be investigated. Interestingly, the fact that we failed to catch migratory individuals using the beach seining method (i.e., fishing in shallow water of <1.5 m), suggests that cod of the frontal ecotype migrate soon after developing past the larval stage. This is supported by data on Atlantic cod juveniles in Sweden where coastal cod juveniles were also more likely to be found in shallower water, with a decrease in abundance with increasing depth (Henriksson et al., 2022).

### 4.3 Implications for fisheries management

Although there is an increasing recognition that personality plays an important role in ecology and population dynamics, it has not much been applied in fisheries management yet (Berger‐Tal et al., 2016; Diaz Pauli & Sih, 2017; Merrick & Koprowski, 2017; Shumway, 1999; Watters et al., 2003). The management of the Icelandic cod stock is currently based on quota, gear selectivity, and temporary fisheries closures (i.e., protected areas), but these management measures/tools are focused on fish size, productivity, and environmental improvement but do not consider fish behavior (Fisheries Management, 2022; Jaworski et al., 2010; Ólafsdóttir & Jakobsdóttir, 2021; Pampoulie et al., 2022).

An often‐suggested method to include behavioral variation into population management is the intended use of mixed‐gear fishing methods that together are unselective for personality type. For example, angling and longlining select for boldness and activity (Härkönen et al., 2014), pots and traps select for boldness and exploration, gill nets select for bold and active individuals, trawlers select for shyness, activity, sociality (MFRI Assessment Reports, 2020), and seining on shyness (Diaz Pauli and Sih (2017) and reference therein). As the *Pan* I locus is part of a supergene located in the LG1 (Berg et al., 2016; Matschiner et al., 2022) containing hundreds of genes maintained by selection processes and strongly discriminating the coastal and frontal behavioral ecotypes, the trend of a correlation of the exploration to the *Pan* I genotype could be representative of the difference in the exploration capacity of the coastal and frontal behavioral ecotypes. If this is the case, then unmanaged use of fishing gear that is unintentionally selected for personality, might deplete one of the behavioral ecotypes and thereby lose migration‐related features, genomic structural variants (Matschiner et al., 2022; Pampoulie et al., 2022), and other variation in the population that could affect population growth and recovery and eventually cause unwanted fisheries‐induced evolution (Árnason et al., 2009; Hutchings et al., 2007; Nusslé et al., 2011; Smith & Blumstein, 2013; Walsh et al., 2006; Ward et al., 2016). It might for example partly explain the *Pan* I genotype fluctuations observed in Icelandic waters (Árnason et al., 2009; Jakobsdóttir et al., 2011). Evidence that size‐selective harvesting can cause a change in available personality types has already been shown in zebrafish (Sbragaglia et al., 2019) and in rainbow trout, where bold and fast‐growing fish were more vulnerable to fisheries (Biro & Post, 2008). Although no direct studies have focused on the relationship between catching method and personality in Atlantic cod, some studies have made efforts to examine how personality influences space use of cod (Villegas‐Ríos et al., 2018), how harvesting targets deep vs shallow water cod (Olsen et al., 2012), how specific gear unintentionally targets cod that behave differently (Bøe, 2014) or are in poor condition (Ovegård et al., 2012), and how cod react to trawling in and in front of the net (Handegard & Tjøstheim, 2005; Rosen et al., 2012). Implementing mixed‐gear methods might also increase the accuracy of estimating the stock biomass, which is commonly underlying quota determination (Morgan, 1997). For example, when fishing methods specifically select bold individuals, it can increase average timidity in the population, and the remaining individuals are therefore harder to catch. This could lead to an underestimation of the total population size (Andersen et al., 2018; Arlinghaus et al., 2017). It is therefore important to include personality in fisheries management. Which specific fishing methods are selecting for the different behavioral ecotypes in Icelandic cod and how these methods need to be arranged remains to be investigated.

Another method used to manage fish populations is the use of MPAs, which aim to protect fish populations against overharvesting and can benefit the fish industry by a spillover of adults, eggs, and larvae beyond the boundaries of the MPAs and into the fished areas. Recent studies suggest that protection by MPAs can affect the behavior of individuals living inside it. For instance, individuals of protected populations typically have a decreased wariness and flight initiation distance compared with fish outside these areas (Bergseth et al., 2016; Januchowski‐Hartley et al., 2015), which might increase the chance of being caught when leaving the protected area (Alós et al., 2015; Diaz Pauli & Sih, 2017). In turn, individual behavior can affect the effectiveness of MPAs. Fish that are more mobile have a higher chance of moving outside the MPA, where their risk of being harvested is higher (de Benito‐Abelló et al., 2022; Dwyer et al., 2020; Mee et al., 2017; Parsons et al., 2010; Pilyugin et al., 2016; Thorbjørnsen et al., 2021; Villegas‐Ríos et al., 2021), especially when fishing pressure on the edges of the reserves is high (Kellner et al., 2007). In this case, if movement behavior has a heritable component, MPAs might cause evolutionary changes within the populations, by favoring resident rather than migratory behavior. This in turn might influence the yield and eventually change the available gene pool in the species (Villegas‐Ríos et al., 2017, 2021). It is therefore important to integrate individual variation in spatial behavior into MPA design and implementation (Claudet et al., 2006; McDermott et al., 2017).

## 5 CONCLUSION

This study shows the first evidence of repeatable exploration behavior and a possible link between this behavior and the migration‐linked *Pan* I locus in the Icelandic cod population, which could have implications for stock management. In a recent study, it was shown that the attribution of *Pan* I to determining migration tendency is more complex than previously assumed. The coastal cod (*Pan* I^AA^) is staying close to the coast as previously thought, but the correlations between the frontal cod genotype (*Pan* I^BB^) and long‐distance migrations are more ambiguous around Iceland (Pampoulie et al., 2022). Further research should therefore move away from solely using the *Pan* I as a determination of migration type when continuing research on personality‐linked stock management.

## AUTHOR CONTRIBUTIONS

**Rosanne Beukeboom:** Conceptualization (equal); data curation (lead); formal analysis (equal); investigation (lead); methodology (lead); software (lead); visualization (lead); writing – original draft (lead); writing – review and editing (lead). **Joseph S. Phillips:** Formal analysis (equal); writing – review and editing (equal). **Guðbjörg Ásta Ólafsdóttir:** Conceptualization (equal); funding acquisition (lead); resources (lead); writing – review and editing (supporting). **David Benhaïm:** Supervision (lead); validation (equal); writing – original draft (supporting); writing – review and editing (equal).

## CONFLICT OF INTEREST STATEMENT

None.

## Data Availability

All data underlying the results in this articles are available at Dryad doi: 10.5061/dryad.79cnp5j07.

## APPENDIX A

**TABLE A1:** Median and 95%uncertainty intervals of the four models (SJ, SM, LA, and LB) invariances of the three personality measurements (OFT, NOT, and MT) and the fixed effects (Allele, SGR, Trial, Test order, and Shelter leave).

SJ	OFT		NOT		MT	
Predictor	Median	UI 95%	Median	UI 95%	Median	UI 95%
*ID level*						
Measurement	0.76	0.46 to 1.31	0.06	0.00 to 0.42	0.19	0.00 to 0.67
Allele (*Pan* I^AA^)	0.42	−0.35 to 1.18	−0.17	−0.48 to 0.81	0.22	−0.44 to 0.86
SGR	0.15	−0.15 to 0.45	−0.14	−0.41 to 0.13	−0.25	−0.52 to 0.01
Trial	0.23	−0.02 to 0.47	−0.16	−0.66 to 0.34	0.12	−0.30 to 0.57
Shelter leave	0.51	0.13 to 0.90	−0.16	−0.73 to 0.41	0.31	−0.26 to 0.88
Object/Mirror order			−0.10	−0.60 to 0.40	0.25	−0.25 to 0.76
*Residual level*						
Measurement	0.2	0.12 to 0.35	1.04	0.71 to 1.51	0.8	0.50 to 1.25
**SM**	**OFT**		**NOT**		**MT**	
**Predictor**	**Median**	**UI 95%**	**Median**	**UI 95%**	**Median**	**UI 95%**
*ID level*						
Measurement	0.58	0.24 to 1.11	0.4	0.06 to 0.92	0.41	0.09 to 0.67
Allele (*Pan* I^AA^)	0.60	−0.14 to 1.34	−0.32	−0.40 to 1.01	0.27	−0.45 to 0.96
SGR	0.19	−0.09 to 0.48	−0.09	−0.36 to 0.18	−0.05	−0.32 to 0.24
Trial	0.18	−0.13 to 0.51	0.02	−0.36 to 0.39	0.07	−0.30 to 0.43
Object/Mirror order	0.31	−0.14 to 0.74	−0.21	−0.69 to 0.28	−0.11	−0.57 to 0.36
Shelter leave			0.17	−0.38 to 0.71	0.46	−0.08 to 1.03
*Residual level*						
Measurement	0.46	0.29 to 0.78	0.69	0.44 to 1.13	0.65	0.42 to 1.05
**LA**	**OFT**		**NOT**		**MT**	
**Predictor**	**Median**	**UI 95%**	**Median**	**UI 95%**	**Median**	**UI 95%**
*ID level*						
Measurement	0.46	0.15 to 0.96	0.20	0.00 to 0.71	0.09	0.00 to 0.50
Allele (*Pan* I^AA^)	0.70	0.02 to 1.39	0.29	−0.35 to 0.92	0.24	−0.36 to 0.82
SGR	0.27	−0.01 to 0.55	−0.25	−0.51 to 0.02	−0.02	−0.26 to 0.23
Trial	−0.05	−0.38 to 0.27	−0.15	−0.57 to 0.24	−0.61	−1.02 to −0.19
Object/Mirror order	0.24	−0.18 to 0.65	−0.05	−0.53 to 0.42	0.46	−0.02 to 0.94
Shelter leave			−0.26	−0.77 to 0.25	0.19	−0.30 to 0.68
Residual level						
Measurement	0.51	0.32 to 0.87	0.83	0.53 to 1.28	0.84	0.55 to 1.26
**LB**	**OFT**		**NOT**		**MT**	
**Predictor**	**Median**	**UI 95%**	**Median**	**UI 95%**	**Median**	**UI 95%**
*ID level*						
Measurement	0.36	0.02 to 0.83	0.02	0.00 to 0.20	0.32	0.00 to 0.91
Allele (*Pan* I^AA^)	0.56	−0.09 to 1.22	0.17	−0.41 to 0.74	0.23	−0.44 to 0.89
SGR	0.21	−0.05 to 0.48	−0.20	−0.44 to 0.05	−0.10	−0.38 to 0.18
Trial	−0.01	−0.43 to 0.40	−0.09	−0.57 to 0.39	−0.43	−0.86 to 0.00
Object/Mirror order	0.21	−0.26 to 0.71	−0.22	−0.76 to 0.29	−0.06	−0.56 to 0.43
Shelter leave			0.38	−0.11 to 0.88	0.36	−0.21 to 0.89
*Residual level*						
Measurement	0.64	0.40 to 1.10	1.01	0.72 to 1.44	0.7	0.41 to 1.20

**TABLE A2:** Median and 95% uncertainty intervals of the covariances of the four models (SJ, SM, LA, and LB) between the pairwise three personality measurements (OFT, NOT, and MT).

SJ		OFT		NOT		MT	
	Predictor	Median	UI 95%	Median	UI 95%	Median	UI 95%
NOT	ID	−0.05	−0.31 to 0.11				
	Residual	0.00	−0.16 to 0.15				
MT	ID	0.04	−0.16 to 0.33	0.00	−0.11 to 0.16		
	Residual	0.08	−0.06 to 0.24	−0.22	−0.50 to 0.03		
**SM**		**OFT**		**NOT**		**MT**	
	**Predictor**	**Median**	**UI 95%**	**Median**	**UI 95%**	**Median**	**UI 95%**
NOT	ID	−0.13	−0.46 to 0.11				
	Residual	−0.10	−0.32 to 0.07				
MT	ID	−0.12	−0.41 to 0.14	0.22	−0.01 to 0.52		
	Residual	0.13	−0.04 to 0.34	−0.08	−0.29 to 0.16		
**LA**		**OFT**		**NOT**		**MT**	
	**Predictor**	**Median**	**UI 95%**	**Median**	**UI 95%**	**Median**	**UI 95%**
NOT	ID	0.01	−0.22 to 0.22				
	Residual	−0.24	−0.49 to −0.04				
MT	ID	−0.01	−0.20 to 0.18	0.02	−0.09 to 0.23		
	Residual	0.06	−0.14 to 0.28	−0.07	−0.32 to 0.17		
**LB**		**OFT**		**NOT**		**MT**	
	**Predictor**	**Median**	**UI 95%**	**Median**	**UI 95%**	**Median**	**UI 95%**
NOT	ID	0.00	−0.13 to 0.12				
	Residual	−0.18	−0.42 to 0.04				
MT	ID	0.05	−0.15 to 0.35	0.01	−0.07 to 0.19		
	Residual	0.08	−0.13 to 0.34	0.04	−0.19 to 0.28		
